# Prognostic Value of Admission Serum Chloride and of the Composition of Metabolic Acidosis in Postoperative Intensive Care: A Single-Centre Retrospective Cohort Study

**DOI:** 10.3390/jcm15218235

**Published:** 2026-10-22

**Authors:** Özkul Yılmaz Çolak, Melda İşevi, Tuğçehan Sezer Akman, Neslihan Ünal Akdemir, Özgür Kılıç

**Affiliations:** 1 Department of Internal Medicine, Division of Intensive Care Medicine, Faculty of Medicine, Ondokuz Mayıs University, 55139 Samsun, Türkiye; ozgur.kilic@omu.edu.tr; 2 Department of Anaesthesiology and Reanimation, Division of Intensive Care Medicine, Faculty of Medicine, Ondokuz Mayıs University, 55139 Samsun, Türkiye; drmeldaisevi@gmail.com (M.İ.); tgchnszr@gmail.com (T.S.A.); neslihan.un.ak@gmail.com (N.Ü.A.)

**Keywords:** chloride, postoperative care, intensive care, ICU mortality, in-hospital mortality, metabolic acidosis, anion gap, strong ion difference, case mix

## Abstract

**Background/Objectives**: Serum chloride is measured in almost every postoperative intensive care unit (ICU) admission. We examined whether it carries prognostic information once operative case-mix and the composition of the metabolic acidosis are accounted for. **Methods**: In 1136 adults admitted from the operating theatre to a single 20-bed ICU, admission chloride was modelled with restricted cubic splines in multivariable logistic regression, and three acid–base features (lactate > 2, chloride > 108, anion gap > 16 mmol/L) were counted in patients with base excess < −3 mmol/L. The primary outcome was ICU mortality. **Results**: A total of 95 patients (8.4%) died in the ICU. Chloride showed no association with ICU mortality on the overall test (χ^2^ = 4.98, 2 df; *p* = 0.083), and its removal left discrimination unchanged (ΔC +0.003, 95% CI −0.004 to +0.021), although the odds ratio for 115 versus 105 mmol/L was 1.91 (1.08–3.38). ICU mortality rose from 6.6% with one acid–base feature to 58.6% with three (Firth’s penalised odds ratio 10.50 (4.65–23.70) versus no metabolic acidosis), a contrast dominated by the depth of the acidosis; adjusted for that depth, each feature carried an odds ratio near 2.0. The anion gap, uncorrected for albumin, and bicarbonate, but not the strong ion difference, were associated with death. **Conclusions**: Admission chloride added no detectable prognostic information, though a modest association is not excluded. Coexisting acid–base abnormalities identified high-risk patients and place the signal in the bicarbonate deficit and unmeasured anions, but this is a descriptive marker needing external validation, not an established risk-stratification tool.

## 1. Introduction

Chloride is among the most frequently measured and least frequently interpreted electrolytes in intensive care. A systematic review of 34 studies and 175,021 critically ill adults reported hyperchloraemia in 34% and hypochloraemia in 14% of patients, with both extremes associated with mortality in a non-linear dose–response relationship [1]. A hospital-wide analysis of more than 76,000 admissions identified 105–108 mmol/L as the range associated with the lowest mortality [2], and in septic patients, a rise in chloride over the first 72 h, rather than the admission value, predicted hospital mortality [3].

These observations come largely from medical and emergency admissions, in whom a deranged chloride plausibly reflects the underlying illness. After surgery, the situation differs. Chloride-rich solutions given during and after the operation raise plasma chloride and lower the strong ion difference [4], producing hyperchloraemic acidosis without any deterioration in the patient [5]. In healthy volunteers, 2 L of 0.9% saline reduced renal artery flow velocity and renal cortical tissue perfusion, whereas the same volume of a balanced solution did not [6]. The acid–base consequence of a given fluid depends on the difference between its strong ion difference and the patient’s pre-infusion bicarbonate, so the same infusion leaves different signatures in different patients [7,8]. A chloride value measured on arrival in the ICU after surgery is therefore, in part, a record of the operation and its fluid regimen.

The evidence in surgical populations is correspondingly discordant. In a mixed ICU cohort, severe hyperchloraemia was independently associated with mortality overall, yet among 2350 patients admitted after elective cardiac surgery, it was present in 86.9% and was not associated with 30-day mortality; if anything, it accompanied lower hospital mortality [9]. In 7991 surgical ICU patients, hyperchloraemia was not associated with postoperative acute kidney injury (odds ratio 1.09, 95% CI 0.80–1.49); only a perioperative rise in chloride, and only in chronic kidney disease stage 3 or worse, was [10]. A propensity-matched cardiac surgical cohort likewise found no association with acute kidney injury, although the magnitude of the chloride rise was independently associated [11]. Where an association has been shown after non-cardiac surgery, it involved postoperative rather than admission chloride, with an absolute risk increase from 1.9% to 3.0% [12]. These are subgroup observations rather than direct tests. The randomised evidence in critically ill adults is itself unsettled: a cluster-randomised trial found a lower rate of the composite of death, new renal-replacement therapy or persistent renal dysfunction with balanced crystalloids [13], whereas two large double-blind trials found no reduction in 90-day mortality [14] or in death or acute kidney injury [15]; an individual-patient-data meta-analysis judged a small reduction in in-hospital mortality probable but of moderate certainty [16], and meta-analytic effects differ by population [17,18]. In the perioperative setting, intraoperative randomisation to balanced crystalloids has an uncertain effect on mortality and renal-replacement therapy but probably yields a higher postoperative pH [19].

Two explanations for this discordance can be separated in a postoperative cohort but rarely are. The first is case-mix: if the prevalence of hyperchloraemia and the risk of death both vary by the type of operation, and vary in different directions, an apparent chloride signal in a mixed postoperative unit can be generated by the composition of the cohort alone. The second is that chloride is not an independent quantity. It enters the anion gap and the strong ion difference by definition, so its prognostic value cannot be assessed without asking which part of the acid–base disturbance carries the information. Metabolic acidosis in critical illness is heterogeneous, and the outcome differs by the predominant anion, being worst in lactic and least severe in hyperchloraemic acidosis [20]. Whether the co-occurrence of several acid–base abnormalities identifies a higher-risk phenotype than isolated abnormalities has not been well characterised after surgery. Any such grouping must be read with its construction in mind: the quantities involved are algebraically linked rather than independent, so combinations of them describe a disturbance rather than constitute candidate prognostic scores, and that is how they are treated here.

Mortality after inpatient non-cardiac surgery is higher than commonly assumed and varies substantially between health systems [21], and postoperative admissions account for a large part of intensive care activity. We therefore examined the association between serum chloride concentration at ICU admission and outcome in consecutive postoperative ICU patients, with ICU mortality as the primary outcome and in-hospital mortality, documented acute kidney injury, and prolonged ICU stay as secondary outcomes. Two questions were addressed beyond the crude association: whether it is explained by operative case-mix, and whether the accompanying acid–base disturbance, decomposed into its components, accounts for it.

## 2. Materials and Methods

### 2.1. Design, Setting and Ethics

This single-centre, retrospective observational cohort study was conducted in the 20-bed mixed medical–surgical intensive care unit of Ondokuz Mayıs University Faculty of Medicine, a tertiary referral hospital in Samsun, Türkiye, and included patients treated between 1 January and 31 December 2024. Ethical approval was obtained from the Ondokuz Mayıs University Clinical Research Ethics Committee (decision no. 2026/513, 10 August 2026). Given the retrospective, non-interventional design of the study, which relied exclusively on pseudonymised routinely collected clinical data and involved no study-specific intervention, additional sampling, or patient contact, individual informed consent was not obtained. The study was conducted in accordance with the Declaration of Helsinki and Turkish Personal Data Protection Law No. 6698 and is reported in accordance with the STROBE statement [22].

### 2.2. Participants

All ICU admissions during the study period were screened. Study-specific eligibility criteria were applied to identify consecutive adult postoperative patients admitted directly to the ICU from the operating theatre. Patients aged 18 years or older were eligible if an admission laboratory set obtained within six hours of ICU admission included serum sodium, potassium, chloride and creatinine, together with arterial pH, bicarbonate, base excess and lactate. The six-hour window was specified in the protocol so that patients whose admission sampling was delayed would not be excluded. When more than one qualifying measurement was available within this window, the earliest value obtained after ICU admission was used for each analyte.

Of the 1423 ICU admissions assessed for eligibility, 146 were excluded because of age < 18 years (*n* = 18), transfer from the ICU of another hospital (*n* = 13), repeat ICU admission (*n* = 8; only the first admission was retained), missing required admission laboratory data (*n* = 31), physiologically implausible laboratory values (*n* = 14), or incomplete or internally inconsistent admission records (*n* = 62). Records were classified as incomplete or internally inconsistent when the date or time of ICU admission could not be reliably established or when the documented chronology of the ICU episode was incompatible with the recorded ICU length of stay. Discrepant entries were cross-checked against the available electronic and clinical records, and records were excluded only when the discrepancy could not be resolved. These exclusions were considered separately from missing required admission laboratory data and physiologically implausible laboratory values.

**Figure 1:**
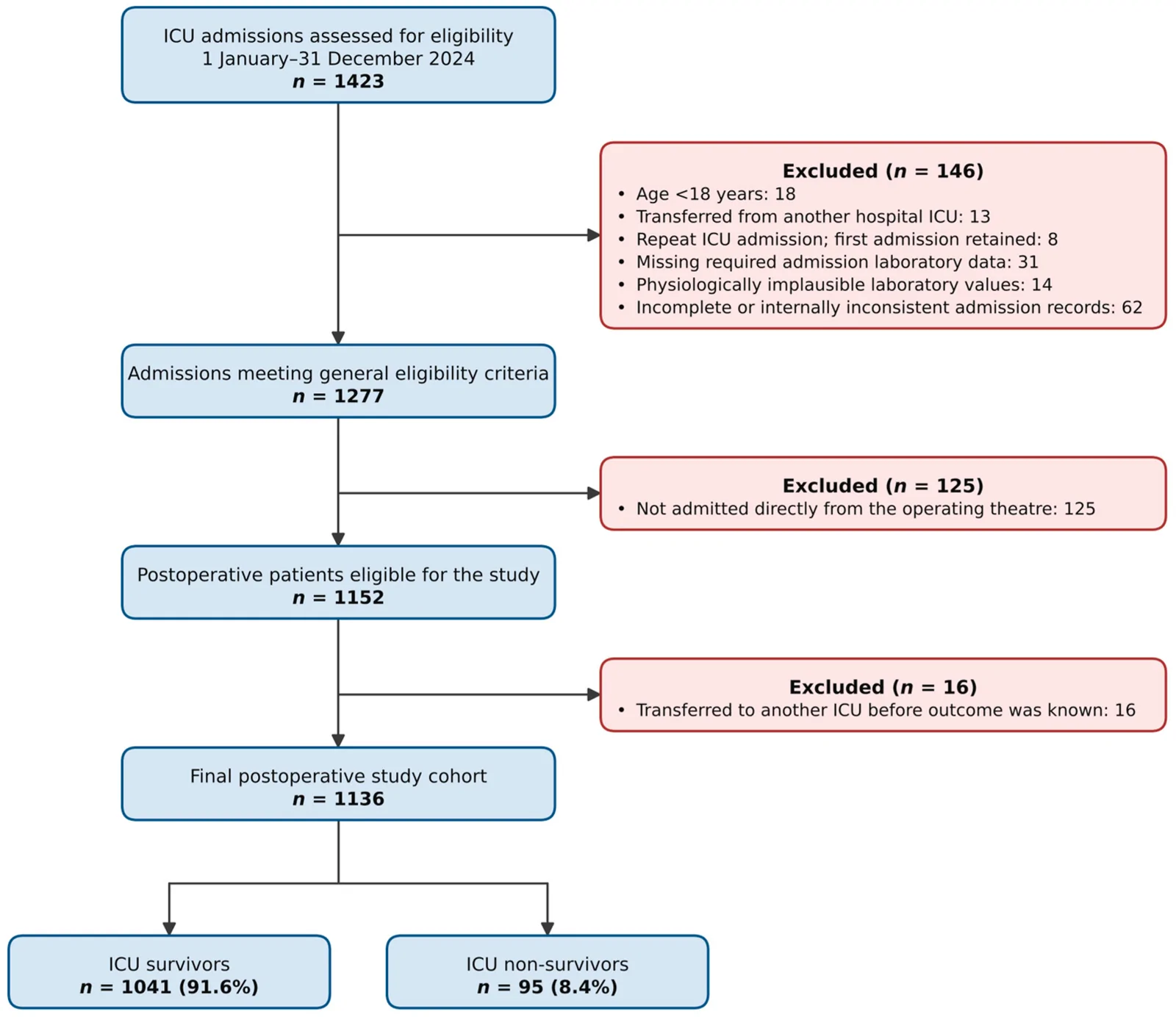
Study flow. Patients transferred to another ICU (*n* = 16) were excluded from the outcome analyses because ICU vital status was unavailable. ICU, intensive care unit.

A further 125 admissions were excluded because ICU admission did not directly follow surgery. Patients who were subsequently transferred to another ICU before the outcome was known (*n* = 16) were excluded from the outcome analyses because ICU vital status could not be ascertained. The final analysed cohort comprised 1136 postoperative patients. The derivation of the analysed cohort is shown in Figure 1.

### 2.3. Data Sources and Measurement

Data were extracted from the hospital information management system, the laboratory information system and the clinical record into a pre-structured collection form, with ambiguous records adjudicated by a second investigator. Identifiers were used only for record linkage, and the analysis dataset was pseudonymised. Sex was retrieved from two independent hospital registry exports, matched by record number; the sources were mutually consistent and together covered the whole cohort.

Unit protocol requires a complete blood count, a biochemistry panel and an arterial blood gas to be drawn together on arrival in the ICU, and both specimens must be obtained within the first 30 min of admission so that the interval between the serum and blood gas specimens contributing to any derived acid–base variable is short. Serum chloride, sodium and potassium were measured in the central hospital biochemistry laboratory on a Roche cobas 8000 analyser (Roche Diagnostics, Mannheim, Germany) by indirect ion-selective electrode potentiometry on diluted serum, and creatinine photometrically on the same platform. Arterial blood gas analysis was performed on a Siemens RAPIDLab 1265 analyser (Siemens Healthineers, Erlangen, Germany), in which bicarbonate and base excess are calculated from the measured pH and carbon dioxide tension rather than measured directly. All chloride values used in this study are laboratory serum values; blood gas chloride was not used, and the two methods were not treated as interchangeable, since indirect and direct potentiometry are not numerically equivalent, particularly at extremes of plasma protein and lipid concentration. The anion gap was therefore calculated from serum sodium, potassium and chloride, together with the blood-gas-derived bicarbonate, and the simplified strong ion difference from serum electrolytes alone; the two specimens are drawn as a single admission set within the 30-minute window described above rather than aliquoted from one sample, so any residual temporal discrepancy is small.

### 2.4. Variables and Outcomes

The exposure was admission serum chloride, analysed continuously and, secondarily, in three pre-specified categories (hypochloraemia < 98, normochloraemia 98–108, hyperchloraemia > 108 mmol/L). These thresholds were fixed in the protocol before analysis: they follow the study laboratory reference interval of 98–108 mmol/L, which contains the 105–108 mmol/L band previously associated with the lowest mortality [2], so the categorical results remain comparable with the existing literature. The continuous spline, which imposes no threshold, is the primary specification, and the categories are secondary. Derived variables were the simplified strong ion difference (sodium + potassium − chloride) and the anion gap (sodium + potassium − chloride − bicarbonate); because the latter includes potassium, the threshold of 16 mmol/L used here corresponds to approximately 12 mmol/L in the potassium-free formulation. Metabolic acidosis was defined as base excess below −3 mmol/L. Albumin was recorded in only 36 of the analysed patients (3.2%), so albumin-corrected anion gap and strong ion gap were not calculated. Measurement was not random. Patients in whom albumin was obtained had a higher Charlson comorbidity index (median 2.5 versus 1.0; *p* = 0.006), a higher anion gap (13.8 versus 12.6 mmol/L; *p* = 0.026) and more often underwent non-elective surgery (44.4% versus 27.2%; *p* = 0.038) than those in whom it was not, and their ICU mortality was numerically higher (16.7% versus 8.1%; *p* = 0.125). The recorded albumin values therefore describe a selected and more severely ill subgroup and cannot be read as the albumin distribution of the cohort.

Within the acidosis group, three predefined acid–base features were recorded: lactate > 2 mmol/L, chloride > 108 mmol/L and anion gap > 16 mmol/L. The acid–base feature count was the number present (0–3). Because patients without metabolic acidosis and acidotic patients with none of the three features are not the same group, the count was analysed as a five-level categorical variable with patients without metabolic acidosis as the reference and, within the acidosis group alone, as an ordinal term; the pooled coding that assigns a count of zero to patients without acidosis is reported alongside these for comparison. Robustness of the gradient to the depth of the acidosis was examined by adding base excess, pH and bicarbonate to the model and by stratifying on base excess. Mutually exclusive phenotypes were defined as no acidosis, pure lactic, pure hyperchloraemic or pure high-anion-gap acidosis (exactly one feature), overlapping (two or three) and unclassified (acidosis without any feature). These are descriptive markers, not independent mechanisms: the anion gap is derived from chloride and bicarbonate, and lactate contributes to the anion gap.

The primary outcome was ICU mortality, defined as death in the ICU as opposed to discharge alive. In-hospital mortality was analysed as a secondary outcome and was defined as death during the index ICU admission, death during any ICU readmission within the study year, or death on the ward after ICU discharge. Readmissions during 2024 were ascertained by matching hospital record numbers across the unit’s admission registers and abstracting the date, indication and outcome of each readmission; a blank readmission field was taken to mean no readmission. Ward deaths were identified from the recorded date of hospital death. The remaining secondary outcomes were acute kidney injury, defined as acute kidney injury documented in the clinical record by the treating team according to the Kidney Disease: Improving Global Outcomes (KDIGO) creatinine and urine output criteria [23] rather than recalculated systematically from serial measurements, and ICU stay exceeding 72 h; both are reported as exploratory, since acute kidney injury events were few and death is a competing event for prolonged stay. Re-intubation, unplanned reoperation, tracheostomy and ICU-acquired infection were recorded but were too infrequent for modelling and are reported descriptively.

### 2.5. Statistical Analysis

Continuous variables are presented as median and interquartile range (IQR) and categorical variables as number and percentage. Baseline characteristics are compared between ICU survivors and non-survivors with the Mann–Whitney U test and the chi-squared test; these comparisons are descriptive and were not used to select covariates. Characteristics across chloride categories are tabulated without significance testing, since the groups are defined by the exposure itself.

The primary analysis related admission serum chloride to ICU mortality using multivariable logistic regression adjusted for age, sex, Charlson comorbidity index [24] (used without its age component, because age entered the model separately), admission lactate, admission creatinine, mechanical ventilation at ICU admission and non-elective surgery. Chloride was modelled as a restricted cubic spline with three knots at the 10th, 50th and 90th percentiles (101, 107 and 113 mmol/L); its overall contribution and its departure from linearity were assessed by likelihood ratio tests against the same model without chloride and against a model with chloride as a linear term. Odds ratios were calculated relative to 105 mmol/L, the lowest point of the fitted curve and a concentration within the lowest-risk range reported in hospital-wide data [2], and the principal contrasts were repeated against the cohort median. Chloride was also analysed in the predefined categories, and adjusted absolute risks were estimated across the observed chloride range by marginal standardisation with 2000 bootstrap resamples. A further model added surgical field as six indicator variables.

The acid–base analyses related the feature count and the mutually exclusive phenotypes to mortality under two adjustment sets: the pre-specified set, which omits admission lactate because lactate elevation is itself one of the defining features, and the same set with lactate added, which is reported alongside it throughout. The three features were also entered simultaneously; models containing base excess, pH or bicarbonate examined whether the feature count merely reflects the depth of the acidosis; and the anion gap, the strong ion difference and bicarbonate were each entered as continuous terms to identify which component of the disturbance carries the prognostic information. Because the three-feature group was small, Firth’s penalised likelihood [25] was used for two separately specified contrasts: three features versus no metabolic acidosis in the five-level analysis and three versus zero features among patients with metabolic acidosis. The reference category is named with each estimate. Interactions between features were exploratory.

Secondary outcomes were analysed with the same covariate sets; because death truncates length of stay, the composite of death or ICU stay longer than 72 h was examined in addition, and a three-level outcome (discharge alive within 72 h, survival with ICU stay beyond 72 h, ICU death) was modelled by multinomial logistic regression so that the two components were separated rather than merged. The chloride association was re-estimated with restricted cubic splines applied to lactate, creatinine and age as well, so that non-linear confounder effects could not be mistaken for an absence of association, and under progressive adjustment from an unadjusted spline to the fully adjusted model; the change in the C statistic and in the Brier score on removing the chloride terms was given a 95% confidence interval from 1000 paired bootstrap resamples in which both models were refitted. Sensitivity analyses treated patients transferred to another ICU as deaths, varied knot placement using the four-knot configuration recommended by Harrell [26], restricted the cohort to elective surgery and excluded patients admitted after trauma or with brain death. Complete-case analysis was used because missing covariate data were minimal, and all tests were two-sided; analyses of secondary outcomes, acid–base phenotypes, interactions and sensitivity analyses were considered exploratory.

Discrimination was summarised by the C statistic, with a 95% confidence interval from 2000 bootstrap resamples and an optimism-corrected estimate from 1000 bootstrap repetitions, and overall predictive accuracy by the Brier score. E-values [27] were calculated to quantify how strong an unmeasured confounder would have to be to account for the association between the acid–base feature count and mortality. E-values are defined on the risk ratio scale, so odds ratios were treated as approximations to risk ratios. This is reasonable at the observed overall mortality of 8.4% but less accurate within the small high-mortality strata, so the E-values are indicative rather than exact.

Analyses were performed in Python 3.11 (Python Software Foundation, Beaverton, OR, USA) with statsmodels 0.15.0 and SciPy 1.17.1 for modelling, pandas 2.3.3 and NumPy 2.4.6 for data management and Matplotlib 3.11.1 for graphics; all of these are freely available open-source libraries with no commercial manufacturer. The analysis code and the derived dataset are available from the corresponding author.

## 3. Results

### 3.1. Study Population

Of 1423 ICU admissions assessed for eligibility, 1136 consecutive postoperative patients were analysed (Figure 1). The median age was 65 years (IQR 53–74); 451 patients (39.7%) were women, and 821 (72.3%) underwent elective surgery. Median admission chloride was 107 mmol/L (IQR 104–110); 38 patients (3.3%) were hypochloraemic, 440 (38.7%) were hyperchloraemic, and 408 (35.9%) had metabolic acidosis on arrival. Median ICU stay was 1.4 days (IQR 1.3–1.9), so most patients were admitted for postoperative monitoring and discharged within two days. In total, 95 patients (8.4%) died in the ICU, and 125 (11.0%) died in hospital. Baseline characteristics according to ICU outcome are shown in Table 1, and characteristics across chloride categories are shown in Appendix A, Table A1.

### 3.2. Admission Chloride and ICU Mortality

**Figure 2:**
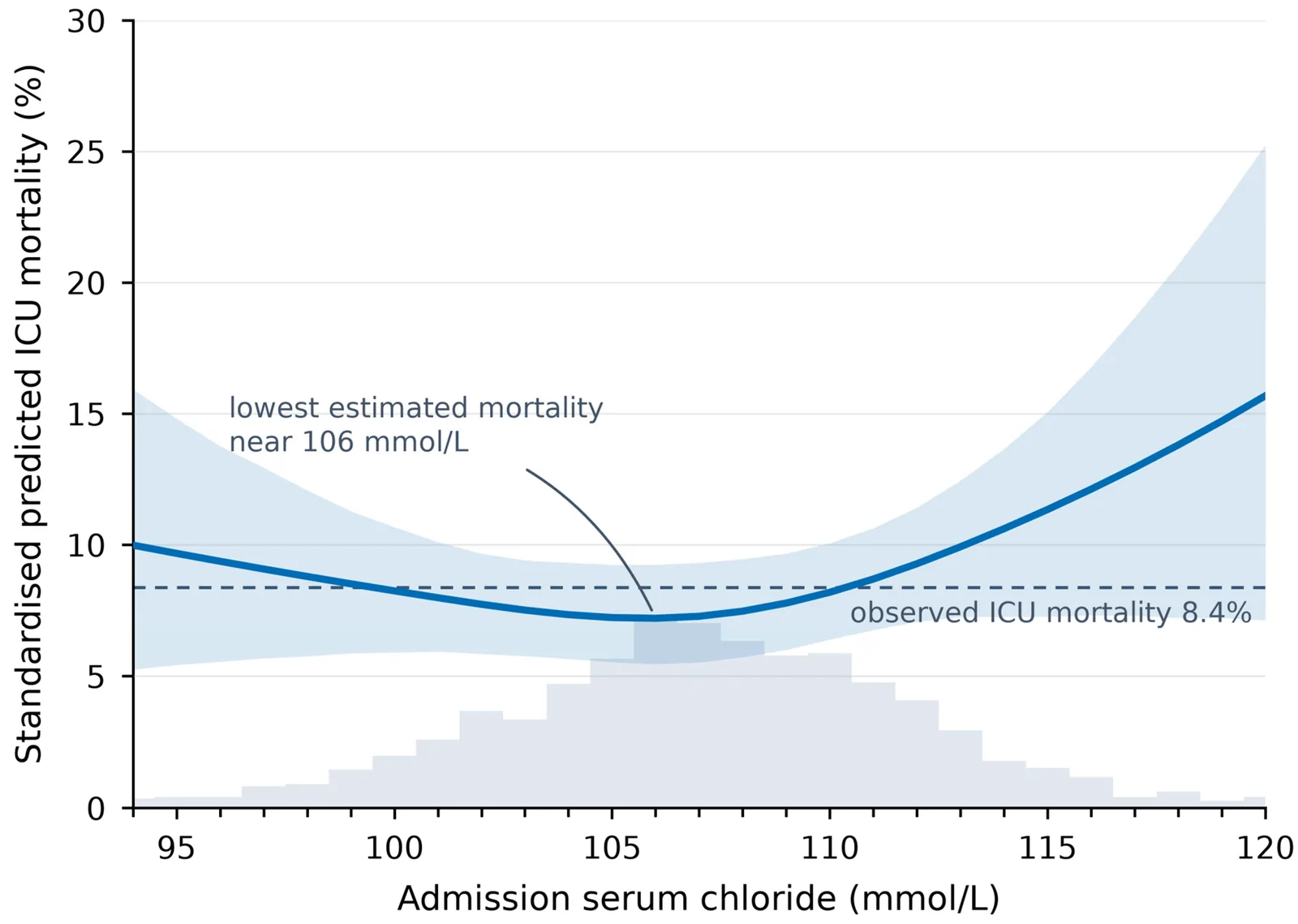
Standardised predicted ICU mortality across the range of admission serum chloride. The solid line shows marginal standardised mortality probabilities from the multivariable model with chloride modelled as a three-knot restricted cubic spline, adjusted for age, sex, Charlson comorbidity index, admission lactate, admission creatinine, mechanical ventilation at ICU admission and non-elective surgery. The shaded band is the 95% bootstrap confidence interval (2000 resamples), the histogram shows the distribution of admission chloride and the dotted lines mark the pre-specified reference range of 98–108 mmol/L. The overall contribution of chloride was not statistically significant (*p* = 0.083), and the nominal departure from linearity (*p* = 0.047) was not robust to knot placement.

In the adjusted model, the overall association between admission chloride and ICU mortality was not statistically significant (likelihood ratio χ^2^ = 4.98, 2 df; *p* = 0.083; Table 2). The fitted curve had its minimum near 105 mmol/L and rose at both extremes, and the test for departure from linearity reached nominal significance (χ^2^ = 3.95, 1 df; *p* = 0.047). This departure was not robust: with the four-knot configuration, it fell to *p* = 0.100, and modelled linearly chloride was null (odds ratio 1.13, 95% CI 0.89–1.42 per 5 mmol/L; *p* = 0.312). The U shape seen in Figure 2 therefore describes the fitted curve in this cohort but should not be presented as an established non-linear association.

On the absolute scale, standardised mortality rose from the curve minimum to 4.1 percentage points higher at 115 mmol/L (95% CI −0.8 to 8.2) and 8.5 percentage points higher at 120 mmol/L (95% CI −1.2 to 18.7); every examined risk difference included zero (Table 3). Repeating the contrasts against the pre-specified cohort median rather than the fitted minimum changed them negligibly (4.1 percentage points for 115 versus 107 mmol/L, 95% CI −0.7 to 8.1), so the presentation does not depend on reading the reference off the curve. With the pre-specified categories, neither hypochloraemia (2.09 (95% CI 0.73–5.98); *p* = 0.170) nor hyperchloraemia (1.25 (95% CI 0.75–2.08); *p* = 0.397) was associated with ICU mortality.

**Figure 3:**
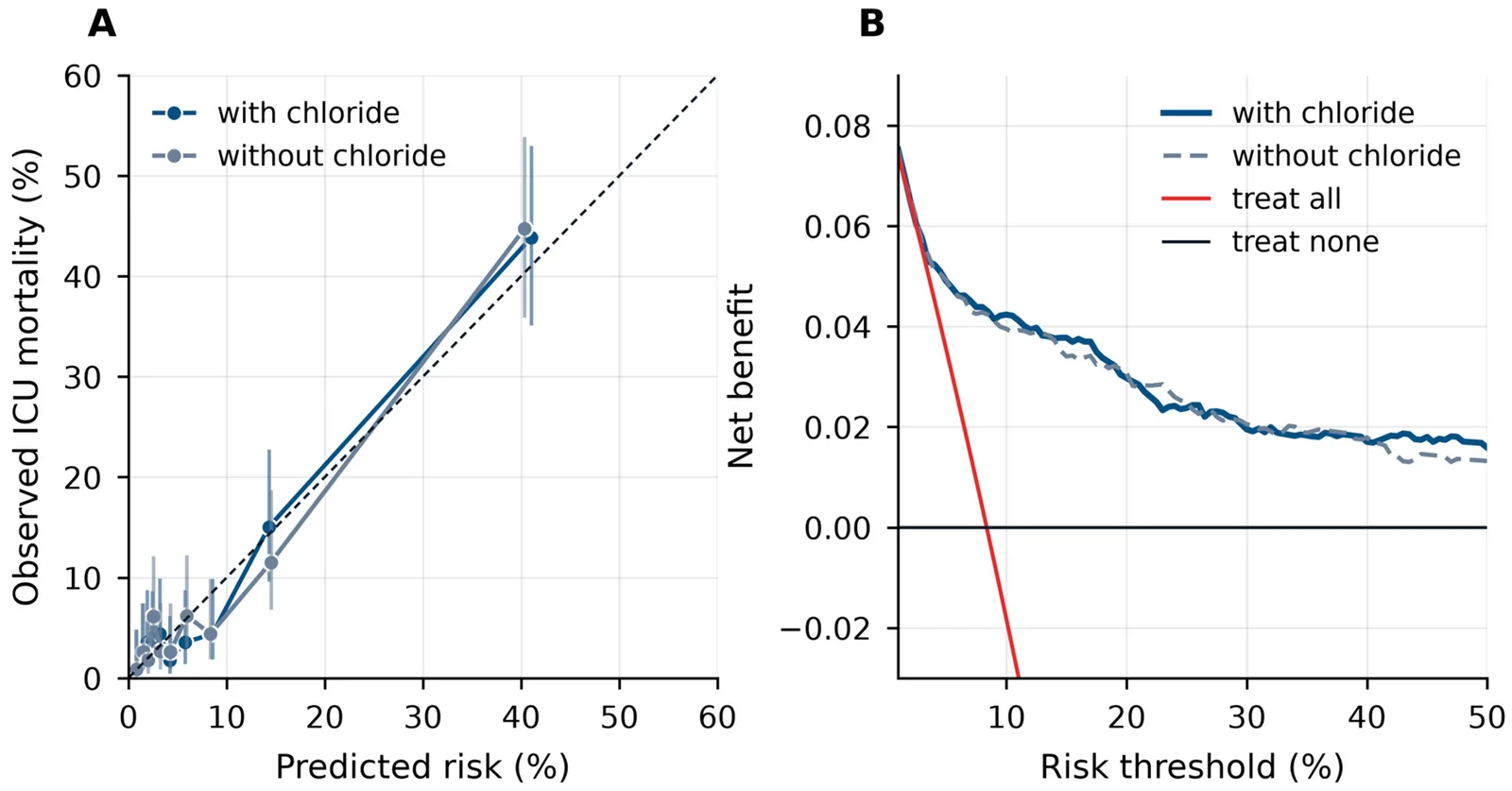
Calibration and clinical utility of the multivariable model. (**A**) Calibration of predicted against observed ICU mortality by decile of predicted risk for the models with and without the chloride spline terms, with the diagonal indicating perfect calibration. (**B**) Decision curves for the same two models across clinically plausible threshold probabilities, together with the treat-all strategy. The two curves are superimposed across the whole range, showing that chloride adds no clinical utility to the model.

The adjusted model discriminated well, with an apparent C statistic of 0.820 (0.780–0.879), an optimism-corrected value of 0.807 and a Brier score of 0.0570. Removing the chloride spline terms left discrimination essentially unchanged (C statistic 0.817; Brier 0.0577; paired bootstrap difference in the C statistic +0.0032, 95% CI −0.0042 to +0.0215), the optimism-corrected C statistic without chloride was 0.807 against 0.807 with it, and calibration was similar for the two models (Figure 3A), whose decision curves were superimposed across the whole range of threshold probabilities (Figure 3B). Admission chloride therefore added no detectable prognostic information beyond the remaining covariates, and the confidence interval for the change in discrimination excludes any gain larger than 0.021.

### 3.3. Chloride, Operative Case-Mix and Mortality

**Figure 4:**
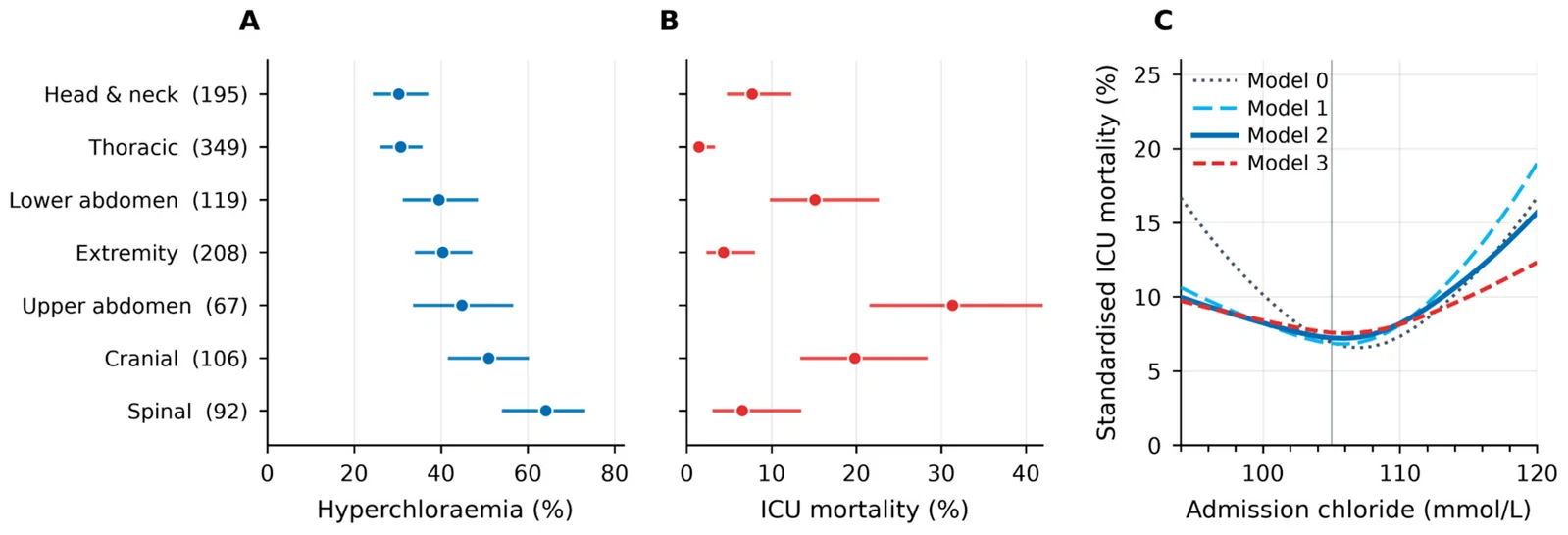
Admission hyperchloraemia and ICU mortality by surgical field. (**A**) Prevalence of admission hyperchloraemia (>108 mmol/L) and (**B**) ICU mortality by surgical field, with 95% Wilson confidence intervals and fields ordered by hyperchloraemia prevalence. (**C**) Standardised predicted ICU mortality across the chloride range under progressive adjustment: chloride alone (Model 0), with patient factors (Model 1), with non-elective surgery, mechanical ventilation and lactate (Model 2) and with surgical field (Model 3). Curves are marginal standardised probabilities; the vertical line marks the 105 mmol/L reference. ICU, intensive care unit.

The prevalence of hyperchloraemia varied more than twofold across surgical fields, from 30.3% after head and neck surgery to 64.1% after spinal surgery, while median admission lactate was nearly identical across fields (Table 4 and Figure 4). This variation did not parallel mortality in any orderly way. Spinal surgery had the highest prevalence of hyperchloraemia with an ICU mortality of 6.5%, whereas upper abdominal surgery had the highest mortality (31.3%) with an intermediate prevalence (44.8%). Across the seven fields, the ordering by hyperchloraemia prevalence did not follow the ordering by mortality (Spearman ρ = +0.32; *p* = 0.48); with only seven fields, this comparison is descriptive and carries no weight beyond the individual-level models.

Adjustment was therefore built up in steps (Table A5 and Figure 4C). Unadjusted, the chloride spline was associated with ICU mortality (χ^2^ = 9.24, 2 df; *p* = 0.010). Adding age, sex, comorbidity index and creatinine did not remove the association (χ^2^ = 7.27, 2 df; *p* = 0.026); adding non-elective surgery, mechanical ventilation and lactate reduced it to *p* = 0.083; and adding surgical field as six indicator variables reduced it further to *p* = 0.320, the odds ratio for 115 versus 105 mmol/L falling from 1.91 (1.08–3.38) in that model to 1.53 (0.84–2.78). The likelihood ratio statistic fell monotonically across the four steps (χ^2^ = 9.24, 7.27, 4.98 and 2.28), the two largest decrements accompanying the introduction of acute severity and of the operation itself, whereas the odds ratio at the upper end of the range varied non-monotonically (Table A5). The association between the acid–base feature count and mortality changed only modestly under the same adjustment (2.61 (95% CI 2.05–3.33) to 2.37 (95% CI 1.83–3.08) per additional feature, on the pooled coding defined in Section 3.4). Because the model contains only 95 deaths, the six indicator variables also reduce precision, so the attenuation is consistent with case-mix but is not on its own decisive. Modelling lactate, creatinine and age with splines as well left the result unchanged (χ^2^ = 4.88, 2 df; *p* = 0.087), so the null finding is not an artefact of assuming linear confounder effects.

### 3.4. Composition of the Acid–Base Disturbance

**Figure 5:**
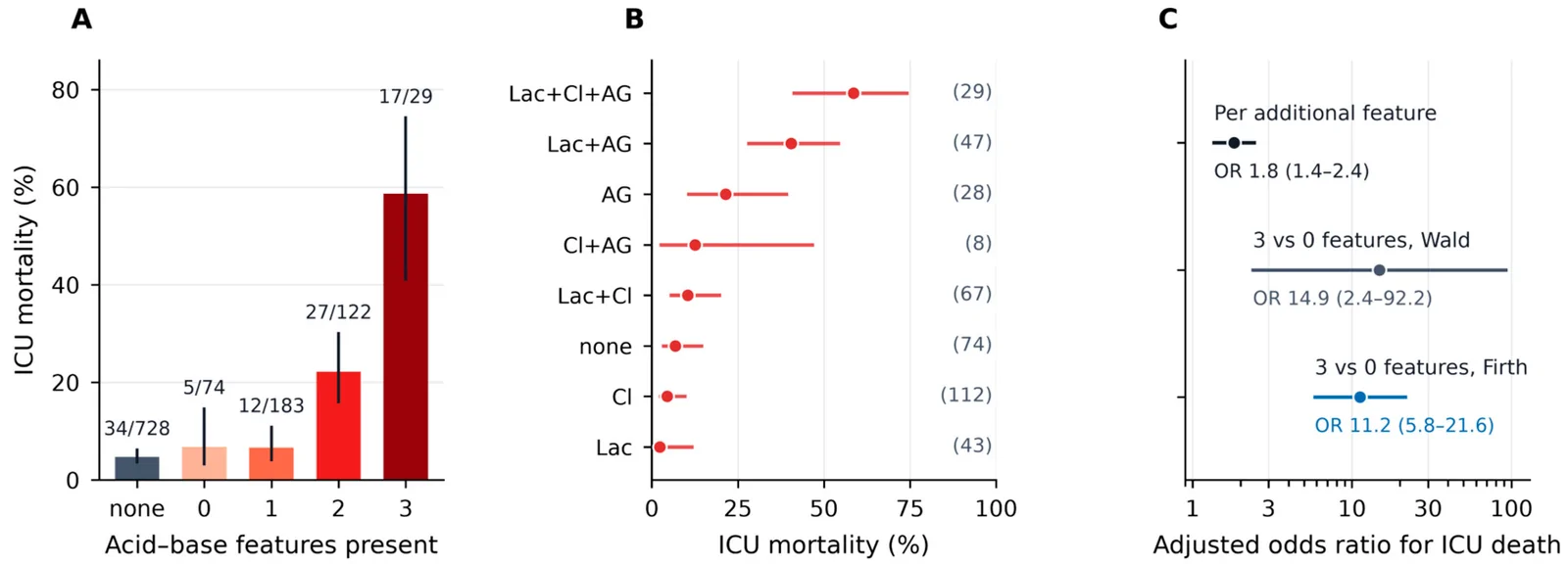
Acid–base abnormalities and ICU mortality. (**A**) ICU mortality by number of acid–base abnormalities, with Wilson 95% confidence intervals. (**B**) Crude mortality for the eight possible feature combinations among patients with metabolic acidosis. (**C**) Adjusted odds ratios for ICU death by mutually exclusive acid–base phenotype, with no metabolic acidosis as reference; models were adjusted for age, sex, Charlson comorbidity index, admission creatinine, mechanical ventilation at ICU admission and non-elective surgery, admission lactate being omitted because it defines one of the features. AG, anion gap; Cl, chloride; Lac, lactate.

ICU mortality increased with the number of coexisting acid–base abnormalities: 4.7% without metabolic acidosis, 6.8% with acidosis but none of the predefined features, 6.6% with one feature, 22.1% with two and 58.6% with all three (Table 5 and Figure 5A,B). Analysed as a five-category variable with patients without metabolic acidosis as the reference, the adjusted odds ratio was 1.56 (0.56–4.36) for acidosis without any of the counted features, 1.10 (0.53–2.27) for one feature, 2.89 (1.44–5.81) for two and 10.97 (3.75–32.10) for three (joint χ^2^ = 21.33, 4 df; *p* = 0.0003); under Firth’s penalisation, which we report in preference, the three-feature contrast was 10.50 (4.65–23.70). Within the acidosis group alone each additional feature carried an adjusted odds ratio of 2.30 (95% CI 1.37–3.85), and the gradient persisted when the depth of the acidosis was added to the model (base excess, 2.01 (95% CI 1.18–3.44); pH, 1.93 (95% CI 1.14–3.29); bicarbonate, 1.86 (95% CI 1.08–3.18)) and within base-excess strata (2.16 (95% CI 0.93–5.00) for −3 to −6 mmol/L and 2.17 (95% CI 1.05–4.48) below −6 mmol/L). Two readings of these numbers should be separated. The contrast between three features and no acidosis is large but is dominated by the severity of the acidosis rather than by the count: a patient carrying all three features is, in practice, a patient with a severe mixed lactic and high-anion-gap acidosis, a state already known to carry high mortality. What survives adjustment for the depth of that acidosis is the smaller and more specific observation that each additional feature retains an odds ratio close to 2.0, that is, that co-occurrence carries information beyond the magnitude of the base deficit. Whether the count adds discrimination beyond the covariates already in the model was not formally assessed, and no such claim is made here. The count is reported as a descriptive marker of how many components of the acid–base disturbance are deranged at once and not as a prognostic score or a risk-stratification tool. The pooled coding, which assigns a count of zero to patients without acidosis, gave 2.61 (95% CI 2.05–3.33) per feature (*p* < 0.001) and 1.82 (95% CI 1.36–2.44) (*p* < 0.001) after adjustment for lactate, which is itself one of the counted features. E-values for the lactate-adjusted estimate were 3.05 for the point estimate and 2.07 for the confidence limit closest to the null, using odds ratios as approximations to risk ratios. On that approximation, an unmeasured confounder would need to be associated with both the feature count and mortality by a risk ratio of about 2.1, beyond the measured covariates, to explain the gradient away. These are sensitivity measures and do not exclude residual confounding by unmeasured acute physiological severity.

The excess risk was concentrated where features overlapped (Figure 5C). Compared with patients without metabolic acidosis, none of the single-feature phenotypes was clearly associated with mortality—pure hyperchloraemic acidosis 0.86 (95% CI 0.31–2.35), pure lactic acidosis 0.42 (95% CI 0.05–3.30), pure high anion gap acidosis 2.62 (95% CI 0.87–7.89)—whereas patients with two or three features had markedly higher mortality (7.87 (95% CI 4.54–13.64); *n* = 151, 44 deaths). Acidosis without any of the predefined features was not associated with mortality (1.34 (95% CI 0.48–3.73)). The contrast between three and zero features takes acidotic patients without any feature as the reference rather than patients without acidosis, rests on 103 patients and is unstable on the Wald scale (14.9 (2.4–92.2)); with Firth’s penalised likelihood, it becomes 11.2 (5.8–21.6), which we report in preference.

Entering the three features simultaneously among patients with metabolic acidosis separated them clearly. An elevated anion gap showed the strongest association with ICU mortality (7.69 (95% CI 3.60–16.43); *p* < 0.001), followed by elevated lactate (3.17 (95% CI 1.48–6.79); *p* = 0.003), while hyperchloraemia was not independently associated (1.89 (95% CI 0.92–3.89); *p* = 0.085). Entering the components as continuous terms pointed the same way: the chloride-containing strong ion difference was null (1.01 (95% CI 0.95–1.07) per mmol/L; *p* = 0.741), whereas the anion gap (1.12 (95% CI 1.05–1.18) per mmol/L; *p* < 0.001) and bicarbonate (0.83 (95% CI 0.77–0.91) per mmol/L; *p* < 0.001) were strongly associated. The anion gap and the simplified strong ion difference share sodium, potassium and chloride, and the anion gap additionally subtracts bicarbonate, so the two results are not independent: the numerical difference between them is exactly bicarbonate. The prognostic information in the admission acid–base panel therefore sits in the bicarbonate deficit and in the unmeasured anion fraction, not in chloride. Because albumin was unavailable in all but 36 patients, the anion gap used throughout is uncorrected. Hypoalbuminaemia lowers the measured gap, so an uncorrected threshold of 16 mmol/L will classify some patients who do have unmeasured anions as normal, and the classification of phenotypes inherits that error. The anion gap results are accordingly reported as hypothesis-generating associations rather than as established prognostic relationships, and the statement that the signal lies in the unmeasured anion fraction is an inference from an uncorrected gap rather than a direct measurement of unmeasured anions.

### 3.5. Secondary Outcomes and Sensitivity Analyses

Acute kidney injury was documented in 36 patients (3.2%), and 149 (13.1%) stayed in the ICU longer than 72 h. The recorded incidence of acute kidney injury is well below that usual in postoperative intensive care and reflects ascertainment from clinical documentation rather than systematic recalculation from serial creatinine and urine output. The endpoint is therefore subject to substantial outcome misclassification, and the estimates that follow are given for completeness only: a null result for an endpoint ascertained in this way is uninformative rather than evidence that no association exists. For documented acute kidney injury, the estimated association with chloride is given for completeness but is not interpretable (0.98 (95% CI 0.92–1.05) per mmol/L, *p* = 0.643); for an ICU stay beyond 72 h, no association with chloride was detected (1.02 (95% CI 0.98–1.06) per mmol/L, *p* = 0.316). The acid–base feature count was associated with both (1.68 (95% CI 1.18–2.41) and 1.36 (95% CI 1.10–1.67) per additional feature). Because death truncates length of stay, the composite of death or stay beyond 72 h was also examined and was the one outcome for which chloride reached significance (χ^2^ = 12.23, 2 df; *p* = 0.002). Modelling the three states separately shows that this is not only an artefact of the competing risk: relative to discharge alive within 72 h, chloride of 115 versus 105 mmol/L was associated both with ICU death (2.31 (95% CI 1.28–4.17)) and with survival beyond 72 h in the ICU (2.11 (95% CI 1.26–3.52); joint χ^2^ = 12.51, 4 df; *p* = 0.014). These are point contrasts at the upper end of the chloride distribution and are reported as exploratory.

Both primary findings held in the restricted cohorts. Among the 821 elective admissions, the overall chloride test remained negative (*p* = 0.490) while each additional acid–base feature still carried an odds ratio of 1.93, and the same held after trauma and brain death were excluded (chloride *p* = 0.391; odds ratio 1.94 per feature). Varying knot placement and the reference concentration, applying Firth’s penalisation and modelling the disturbance continuously left the conclusions unchanged (Appendix A, Table A2).

### 3.6. In-Hospital Mortality and ICU Readmission

Extending the outcome beyond ICU discharge added 30 deaths: 27 of the 68 patients readmitted to the ICU during 2024 died during the readmission, and 3 died on the ward without ICU readmission, giving 125 in-hospital deaths (11.0%) against 95 ICU deaths (8.4%). Mortality among readmitted patients was 39.7%, and it was highest when the indication was respiratory or septic (Appendix A, Table A3).

**Figure 6:**
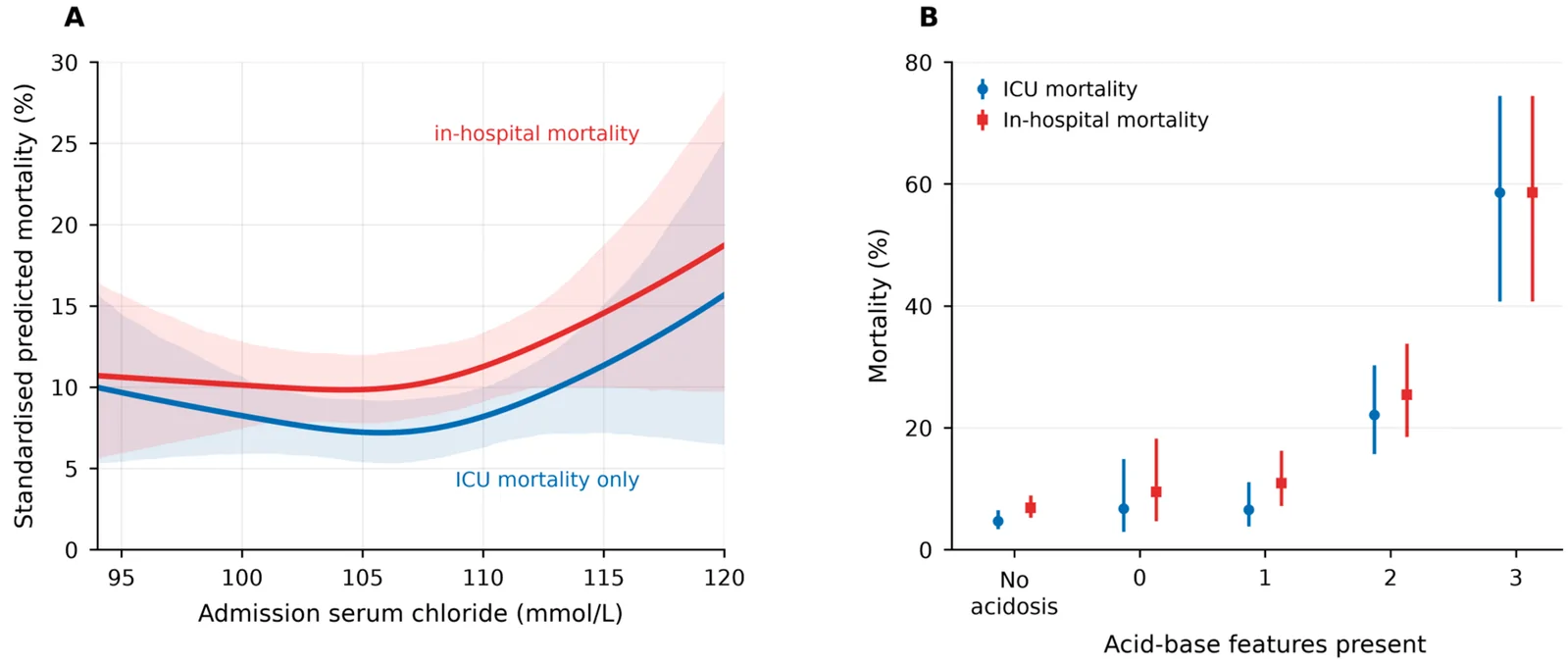
ICU mortality and in-hospital mortality compared. (**A**) Marginal standardised mortality across the range of admission chloride for the two outcomes, from identical model specifications, with 95% bootstrap confidence bands (2000 resamples). (**B**) Observed mortality by acid–base feature count for the two outcomes with binomial 95% confidence intervals. The vertical distance between the curves in (**A**) is the mortality occurring after ICU discharge; its near constancy across the chloride range indicates that post-ICU deaths are not concentrated at either chloride extreme.

The two outcomes behave differently for chloride and show similar patterns for the acid–base features (Table 6). For chloride, the overall test remained non-significant (χ^2^ = 4.16, 2 df, *p* = 0.125), and the nominal departure from linearity disappeared (χ^2^ = 1.82, 1 df, *p* = 0.177), while the hyperchloraemic contrasts kept their direction and significance (1.75 (1.04–2.93) at 115 mmol/L and 2.56 (1.06–6.17) at 120 mmol/L; Figure 6A). Discrimination was lower for the wider outcome and was similar with and without chloride (C statistics 0.796 and 0.793 respectively), indicating no detectable incremental improvement. The acid–base gradient persisted: 2.27 (95% CI 1.82–2.82) per additional feature on the pre-specified covariate set and 1.66 (95% CI 1.28–2.16) with lactate added, with an E-value of 2.71 (Figure 6B and Appendix A, Table A4). The single-feature phenotypes remained null, and the overlapping phenotype remained strongly associated (5.96 (95% CI 3.62–9.82)). The composition of the acid–base disturbance therefore predicts death wherever in the hospital it occurs, whereas the apparent non-linearity of chloride does not survive a change of outcome definition.

## 4. Discussion

In 1136 consecutive postoperative ICU patients, admission serum chloride showed no association with mortality on the overall test of the spline, added no detectable improvement in model discrimination or clinical utility. The documented acute kidney injury endpoint was too poorly ascertained to contribute evidence in either direction and is not relied upon here. Point contrasts at the upper end of the range were compatible with a modest increase in risk, and in a three-level outcome model, chloride was associated with prolonged ICU stay among survivors as well as with ICU death, so the finding is better described as an absence of independent prognostic value than as proof of no association. The apparent U shape of the chloride–mortality curve, which is the finding most often reported in medical cohorts, was reproduced here but proved fragile: it did not survive a change in knot placement, and it disappeared when the outcome was extended to in-hospital death. Two features of the cohort explain why an admission chloride value behaves this way after surgery. Hyperchloraemia varied more than twofold across surgical fields while mortality varied in a different order, so case-mix alone can generate an apparent chloride signal in a mixed postoperative unit; and when the acid–base disturbance was decomposed, the prognostic information sat in the bicarbonate deficit and the unmeasured anion fraction rather than in chloride. What did predict death, consistently and across both outcome definitions, was the coexistence of several acid–base abnormalities.

Previous studies in broader critically ill populations have described a U-shaped association between serum chloride and mortality, with the lowest risk within or close to the normal range [1,2]. We observed a similar shape, and it is worth being explicit about how much weight it can bear. The non-linearity test interrogates only the shape of the fitted curve, so it can reach nominal significance while the overall contribution of the variable remains within what chance would produce, which is what happened here. Two further checks point the same way: with a four-knot configuration, the departure from linearity was no longer significant, and with in-hospital rather than ICU death as the outcome, it disappeared altogether. The safest reading is that the shape resembles the one described in medical populations but that its magnitude here is small and its statistical support weak. Hypochloraemia was uncommon in our cohort, and the higher crude mortality of these patients was no longer evident after adjustment, suggesting that a low chloride value also travels with other clinical features rather than acting independently.

Our findings are consistent with the surgical literature. Hyperchloraemia was not associated with higher mortality after elective cardiac surgery despite being present in the great majority of patients [9], and studies in surgical ICU and cardiopulmonary bypass populations have not shown a consistent association with acute kidney injury, although in the same cohorts, the perioperative rise in chloride was associated with outcome even when the absolute concentration was not [10,11]. An association between postoperative hyperchloraemia and adverse outcome has been reported after non-cardiac surgery [12]. We could not examine perioperative chloride change because only the admission value was available, and this is the more likely locus of any real effect: a single arrival value conflates the patient’s pre-operative state with the intraoperative fluid regimen, whereas a change over time removes the former.

The case-mix analysis makes that conflation visible. Hyperchloraemia was roughly twice as common after spinal as after head and neck surgery, whereas admission lactate was nearly identical across fields—a pattern more easily explained by differences in operative duration and fluid administration than by differences in acute physiological severity. This remains an inference from the pattern of the data and not a demonstration: neither operative duration nor the type and volume of fluid given was recorded, so the analysis establishes that admission chloride varies systematically with the operation without identifying what produces that variation. Mortality, meanwhile, ranged from 1.4% to 31.3% across the same fields and in a different order, and adjustment for surgical field attenuated the chloride estimates while leaving the acid–base gradient essentially intact. Since both the exposure prevalence and the risk of death vary by field, and vary independently of one another, a chloride association estimated in a mixed postoperative cohort without accounting for the operation is open to confounding by the operation itself. This is a structural point rather than a feature of our data, and it applies to any mixed surgical ICU cohort in which admission chloride is interpreted.

The acid–base findings provide the positive counterpart. Outcome in metabolic acidosis is known to differ by predominant anion, with lactic and unmeasured-anion acidosis carrying a worse prognosis than hyperchloraemic acidosis [20], and the prognostic value of the anion gap and strong ion gap has been described in surgical and other critically ill populations [28,29,30]. Our cohort adds two things. First, the risk is carried by the coexistence of abnormalities rather than by any of them alone: no single-feature phenotype was associated with mortality, whereas patients with two or three features had markedly higher mortality, and the gradient persisted under adjustment for lactate, for the depth of the acidosis and within strata of base excess. The composition of an acidosis therefore carries information beyond its magnitude. That statement should be read narrowly. The three features are not independent quantities: the anion gap is computed from chloride and bicarbonate, lactate is itself an unmeasured anion that raises the gap, and the construct is conditioned throughout on a base excess below −3 mmol/L, so a patient who accumulates all three is close to the definition of a severe mixed lactic and high-anion-gap acidosis. The count is best understood as a compact description of how many components of the disturbance are deranged at once, rather than as an additive score in which each feature contributes an independent increment of risk. Consistent with that reading, we make no claim that the count supplies prognostic information beyond the clinical and laboratory variables already available on admission; its incremental predictive performance was not assessed here and would have to be demonstrated before the count could be put to any prognostic use. Second, when the disturbance is decomposed into continuous terms, the chloride-containing strong ion difference is null while the anion gap and bicarbonate are strongly associated. These variables are algebraically linked and should not be read as independent biological mechanisms, but the asymmetry is informative: it locates the prognostic signal in the bicarbonate deficit and the unmeasured anions rather than in chloride, which is exactly what a case-mix interpretation of admission hyperchloraemia predicts.

Extending the outcome from ICU to in-hospital death was also a test of these two readings, and they behaved as expected. The acid–base gradient persisted with a similar magnitude and a similar E-value, whereas the chloride non-linearity vanished. That consistency does not exclude residual confounding by unmeasured physiological severity: an E-value states the strength a confounder would need, not that none is present. A finding that depends on where in the hospital a patient dies is not a robust prognostic marker; a finding that does not is closer to reflecting the state of the patient on arrival.

Taken together, these results do not support interpreting an isolated elevation of admission chloride as an independent marker of poor postoperative outcome. Chloride is more informative when it is read together with lactate, the anion gap and the overall acid–base disturbance than when it is read alone. This should not be taken as evidence for or against any particular intravenous fluid strategy, since neither the type nor the volume of perioperative fluid was recorded, and the randomised comparisons of balanced with chloride-rich crystalloids summarised above remain divided [13,14,15,16,19].

The composition of the cohort should be kept in view when these findings are applied elsewhere. The median ICU stay was 1.4 days, and surgery was elective in 72.3%, so most patients were admitted for postoperative monitoring and discharged within two days, while mortality was concentrated in a minority and differed markedly by surgical field. The cohort therefore behaves less like one population than like several, assembled by the shared route of admission directly from the operating theatre. This structure is typical of mixed postoperative units and is the setting in which an admission chloride value is most often interpreted, but it differs from the medical and emergency populations in which the chloride–mortality association was first described, and the present estimates should not be transferred to units admitting predominantly emergency surgical or medical patients.

The study has several strengths. It included consecutive postoperative ICU admissions over a complete year with near-complete outcome ascertainment, and outcomes were followed beyond ICU discharge to hospital death, including deaths during ICU readmission. Chloride was analysed continuously without imposing a threshold and reported on both relative and absolute risk scales. The robustness of each principal claim was tested rather than asserted: knot placement and reference choice for the spline, Firth’s penalisation for the sparse three-feature contrast, E-values for unmeasured confounding, a three-level outcome model separating death from prolonged stay, and a second outcome definition. Calibration and decision-curve analysis were used alongside discrimination so that the absence of added value from chloride is shown on a clinical as well as a statistical scale.

Several limitations should be considered. First, this was a retrospective single-centre study, and residual confounding cannot be excluded. Established severity scores such as APACHE II and SOFA and information on vasoactive support were unavailable, so the models do not fully capture acute physiological and haemodynamic severity. Two observations bear on this, though neither resolves it. The adjustment set discriminated well, with an apparent C statistic of 0.820, within the range reported for established severity scores in postoperative cohorts; good discrimination is, however, a property of prediction rather than evidence of adequate confounder control, and the two should not be conflated. For the acid–base findings, the E-values state what would be required of an unmeasured confounder: an association with both the feature count and mortality of at least a risk ratio of 2.1, over and above the measured covariates. An E-value quantifies the strength such a confounder would need; it does not show that none is present, and acute physiological severity, which is precisely what is missing from these models, could plausibly reach that strength in a critically ill population. Residual confounding by severity therefore remains a live possibility for every association reported here, and the acid–base findings in particular should be read as associations observed in the absence of a severity score rather than as demonstrably independent of severity. The primary model estimated nine parameters for 95 deaths, approximately ten events per parameter, which is at the lower end of what is usually considered adequate; the optimism-corrected C statistic and the penalised-likelihood re-estimation of the three-feature contrast are reported for that reason. Second, neither the type nor the volume of intravenous fluid given during and after the operation was recorded. Chloride load is the mechanism through which the exposure is usually held to act, and an equivalent volume of saline and of a balanced solution have measurably different renal haemodynamic effects [6], so a null result for the admission concentration cannot be read as evidence that chloride load is harmless. What this cohort measures is the prognostic value of a single value obtained on arrival in the ICU, not the effect of perioperative chloride exposure; the contribution of perioperative management to the observed concentrations is inferred from differences across surgical fields rather than demonstrated directly. Third, chloride was measured only at ICU admission, and perioperative change could not be evaluated; chloride was measured by indirect potentiometry, so the numerical thresholds used here do not transfer directly to direct-potentiometry measurements. Only 38 patients were hypochloraemic, so the low end of the distribution, where an association with mortality has been reported in other critically ill populations [31], is poorly represented here. Fourth, preoperative chloride, the timing of individual laboratory samples and serum total carbon dioxide were not available in the data extract, so the perioperative change in chloride, time-resolved analyses and a bicarbonate-independent check of the acid–base classification could not be performed.

Fifth, albumin was available in only 36 of the analysed patients, so neither an albumin-corrected anion gap nor a strong ion gap could be calculated. This bears directly on the anion gap findings. Each 1 g/dL fall in albumin lowers the measured anion gap by approximately 2.5 mmol/L, and hypoalbuminaemia is common after major surgery, so an uncorrected gap classifies some patients with unmeasured anions as normal; the strong ion gap and the albumin- and lactate-corrected anion gap have been compared directly in septic intensive care patients [32]. The direction of the resulting bias is uncertain, because hypoalbuminaemia after major surgery is itself associated with outcome and may therefore be related to both the measured gap and mortality. Albumin was also not measured at random: it was obtained in patients with greater comorbidity, a higher anion gap and more non-elective surgery, so the recorded values describe a selected subgroup and cannot be taken as the albumin distribution of the cohort, which remains unknown. The anion gap findings should accordingly be regarded as hypothesis-generating rather than as established associations, and the 16 mmol/L threshold should not be transferred to cohorts with a different albumin distribution without correction.

Sixth, acute kidney injury was identified from clinical documentation according to KDIGO criteria rather than recalculated from serial creatinine and urine output and is therefore likely to have been under-ascertained. A recorded incidence of 3.2% is implausibly low for postoperative patients requiring intensive care and indicates substantial outcome misclassification rather than a genuinely low event rate. We therefore treat this endpoint as uninterpretable and draw no conclusion from the absence of an association between chloride and acute kidney injury; that question remains open rather than answered here. Seventh, ICU readmissions were ascertained within the study year and from this hospital only, so a patient readmitted elsewhere or after 31 December 2024 would be misclassified as a survivor; the in-hospital mortality figure is thus a lower bound, as are ward deaths in patients never readmitted to the ICU. Finally, several phenotype groups contained few deaths, so the analyses of individual phenotypes and of interactions are exploratory and require external validation, preferably in cohorts with albumin-corrected acid–base data and recorded perioperative fluid exposure.

## 5. Conclusions

In postoperative intensive care patients, admission serum chloride carried no detectable prognostic information: it showed no association with ICU or in-hospital mortality on the overall tests, and adding it to a multivariable model improved neither discrimination nor clinical utility. This is an absence of convincing evidence for incremental prognostic value rather than evidence that no clinically relevant association exists; the confidence interval for the change in discrimination still admits a gain of up to 0.021, and the documented acute kidney injury endpoint was too poorly ascertained to contribute either way. Point contrasts at the upper end of the chloride range, and a three-level model in which chloride was associated with prolonged ICU stay among survivors as well as with ICU death, leave a modest association possible. The U-shaped curve reported in medical populations was reproduced but was not robust to knot placement or to the outcome definition, and it attenuated further when operative case-mix was taken into account. What identified patients at substantially higher risk was the coexistence of several acid–base abnormalities, an association that persisted under adjustment for lactate and for the depth of the acidosis and held for in-hospital as well as ICU death. The three features are algebraically and physiologically interdependent, and their co-occurrence marks a severe mixed acidosis; their incremental predictive performance was not assessed and severity of illness was not measured. This is therefore a potentially useful risk indicator that requires external validation, not an established approach to postoperative risk stratification. Because the anion gap and the strong ion difference differ only by bicarbonate, decomposing the disturbance places the prognostic information in the bicarbonate deficit and the unmeasured anion fraction rather than in chloride itself. That decomposition rests on an anion gap that could not be corrected for albumin and is offered as a hypothesis about where the signal lies rather than as a settled finding. Admission chloride should therefore be read as part of the acid–base and perioperative picture rather than as an isolated marker of postoperative risk. Confirmation in prospective multicentre cohorts with perioperative fluid data, serial chloride measurements and albumin-corrected acid–base variables is required.

## Figures and Tables

**Table 1:** Baseline characteristics according to ICU outcome.

Characteristic	All Patients (*n* = 1136)	ICU Survivors (*n* = 1041)	ICU Deaths (*n* = 95)	*p* Value
Patients, *n*	1136	1041	95	
Age, years, median (IQR)	65 (53–74)	65 (52–74)	69 (58–76)	0.006
Charlson comorbidity index, median (IQR)	1 (0–2)	1 (0–2)	2 (0–4)	<0.001
Sodium, mmol/L, median (IQR)	138 (136–140)	138 (136–140)	138 (135–142)	0.364
Potassium, mmol/L, median (IQR)	4.4 (4.0–4.8)	4.4 (4.0–4.8)	4.4 (3.9–5.0)	0.637
Chloride, mmol/L, median (IQR)	107 (104–110)	107 (104–110)	107 (103–111)	0.921
Bicarbonate, mmol/L, median (IQR)	23.0 (21.0–24.0)	23.0 (21.0–24.0)	20.0 (16.0–22.6)	<0.001
pH, median (IQR)	7.36 (7.31–7.41)	7.36 (7.32–7.41)	7.30 (7.19–7.38)	<0.001
Base excess, mmol/L, median (IQR)	−1.8 (−4.0 to 0.4)	−1.6 (−3.8 to 0.5)	−5.2 (−10.2 to −1.9)	<0.001
Lactate, mmol/L, median (IQR)	1.7 (1.2–2.3)	1.6 (1.2–2.2)	2.5 (1.5–3.9)	<0.001
Creatinine, mg/dL, median (IQR)	0.88 (0.70–1.12)	0.86 (0.70–1.10)	1.30 (0.91–2.15)	<0.001
Albumin, g/dL, median (IQR) (*n* = 36: 30 survivors, 6 deaths)	2.9 (2.4–3.3)	2.9 (2.5–3.4)	2.4 (2.1–2.5)	0.032
Anion gap, mmol/L, median (IQR)	12.6 (10.2–15.0)	12.4 (10.0–14.6)	16.6 (12.9–21.0)	<0.001
Apparent strong ion difference, mmol/L, median (IQR)	35.3 (33.0–37.6)	35.2 (33.0–37.5)	36.4 (33.9–39.3)	0.004
ICU stay, days, median (IQR)	1.4 (1.3–1.9)	1.4 (1.3–1.6)	4.3 (1.3–8.4)	<0.001
Female sex, *n* (%)	451 (39.7)	412 (39.6)	39 (41.1)	0.864
Non-elective surgery, *n* (%)	315 (27.7)	258 (24.8)	57 (60.0)	<0.001
Mechanically ventilated on arrival, *n* (%)	1016 (89.4)	927 (89.0)	89 (93.7)	0.218
Trauma, *n* (%)	66 (5.8)	58 (5.6)	8 (8.4)	0.364
Metabolic acidosis (base excess < −3 mmol/L), *n* (%)	408 (35.9)	347 (33.3)	61 (64.2)	<0.001
Documented acute kidney injury, *n* (%)	36 (3.2)	14 (1.3)	22 (23.2)	<0.001
ICU stay > 72 h, *n* (%)	149 (13.1)	97 (9.3)	52 (54.7)	<0.001
Thoracic, *n* (%)	349 (30.7)	344 (33.0)	5 (5.3)	<0.001
Extremity, *n* (%)	208 (18.3)	199 (19.1)	9 (9.5)	
Head and neck, *n* (%)	195 (17.2)	180 (17.3)	15 (15.8)	
Lower abdominal, *n* (%)	119 (10.5)	101 (9.7)	18 (18.9)	
Cranial, *n* (%)	106 (9.3)	85 (8.2)	21 (22.1)	
Spinal, *n* (%)	92 (8.1)	86 (8.3)	6 (6.3)	
Upper abdominal, *n* (%)	67 (5.9)	46 (4.4)	21 (22.1)	

Data are median (interquartile range) or *n* (%). *p* values are from the Mann–Whitney U test for continuous and the chi-squared test for categorical variables and are descriptive. The Charlson comorbidity index was used without its age component in multivariable models, with age entered separately. Albumin was measured in only 36 of the 1136 patients (3.2%) and, as described in Section 2.4, in a selected subgroup with greater comorbidity, a higher anion gap and more non-elective surgery; the albumin row and its *p* value therefore describe that subgroup and not the cohort, and they are retained only to show that measured concentrations were low where albumin was obtained. BE, base excess; ICU, intensive care unit; IQR, interquartile range.

**Table 2:** Admission chloride and ICU mortality: multivariable model.

Term	OR	*p*
Admission chloride, restricted cubic spline (101/107/113 mmol/L)	-	LR χ^2^ = 4.98, 2 df, *p* = 0.083
Departure from linearity	-	LR χ^2^ = 3.95, 1 df, *p* = 0.047
Age, per year	1.03 (1.01–1.04)	<0.001
Female sex	1.16 (0.70–1.92)	0.576
Charlson comorbidity index, per point	1.22 (1.09–1.36)	<0.001
Admission lactate, per mmol/L	1.70 (1.47–1.96)	<0.001
Admission creatinine, per mg/dL	1.49 (1.22–1.82)	<0.001
Mechanical ventilation on arrival	2.24 (0.90–5.58)	0.082
Non-elective surgery	4.75 (2.89–7.81)	<0.001
C statistic (apparent)	0.820 (0.780–0.879)	
C statistic (bootstrap optimism-corrected, 1000 replicates)	0.807	
C statistic, model without chloride	0.817	
Brier score, with/without chloride	0.0570/0.0577	

Multivariable logistic regression adjusted for the listed covariates, with chloride modelled as a three-knot restricted cubic spline. The overall test compares this model with the same model without chloride; the departure-from-linearity test compares it with a model containing chloride as a linear term. The C statistic confidence interval is from 2000 bootstrap resamples and the optimism correction from 1000 Harrell bootstrap repetitions. CI, confidence interval; LR, likelihood ratio.

**Table 3:** Adjusted odds ratios, standardised absolute risk differences and pre-specified chloride categories.

Chloride, mmol/L	Adjusted OR Versus 105 mmol/L	Risk Difference Versus 105 mmol/L, pp	Risk Difference Versus Median, pp
92	1.74 (0.65–4.63)		
95	1.51 (0.72–3.19)	+2.4 (−2.1 to +7.7)	+2.4 (−2.5 to +8.0)
100	1.20 (0.84–1.72)	+1.0 (−1.1 to +3.0)	+1.0 (−1.6 to +3.2)
105	1.00 (reference)	0 (reference)	
110	1.19 (0.96–1.48)	+1.0 (−0.5 to +2.1)	+0.9 (−0.2 to +1.7)
115	1.91 (1.08–3.38)	+4.1 (−0.8 to +8.2)	+4.1 (−0.7 to +8.1)
120	3.11 (1.18–8.19)	+8.5 (−1.2 to +18.7)	+8.4 (−1.2 to +18.7)
Hypochloraemia < 98 mmol/L	2.09 (0.73–5.98)		
Hyperchloraemia > 108 mmol/L	1.25 (0.75–2.08)		

Odds ratios and risk differences come from the model in Table 2. Risk differences are marginal standardised absolute differences in percentage points with 2000-resample bootstrap confidence intervals; the median column uses the pre-specified cohort median of 107 mmol/L as reference. The final two rows use the pre-specified categories with normochloraemia (98–108 mmol/L) as reference. OR, odds ratio; pp, percentage points.

**Table 4:** Admission chloride, hyperchloraemia and mortality by surgical field.

Surgical Field	*n*	Median Chloride, mmol/L	Median Lactate, mmol/L	Hyperchloraemia, %	ICU Deaths, *n* (%)	In-Hospital Deaths, *n* (%)
Head and neck	195	106	1.7	30.3	15 (7.7)	18 (9.2)
Thoracic	349	106	1.6	30.7	5 (1.4)	12 (3.4)
Lower abdominal	119	107	1.8	39.5	18 (15.1)	22 (18.5)
Upper abdominal	67	107	2.1	44.8	21 (31.3)	21 (31.3)
Extremity	208	108	1.6	40.4	9 (4.3)	18 (8.7)
Cranial	106	109	1.7	50.9	21 (19.8)	27 (25.5)
Spinal	92	110	1.6	64.1	6 (6.5)	7 (7.6)

Fields are ordered by median admission chloride. Hyperchloraemia was defined as admission serum chloride > 108 mmol/L. Percentages for deaths are within field. Multivariable models adding surgical field as six indicator variables are described in the text. ICU, intensive care unit.

**Table 5:** Acid–base phenotype, feature count and ICU mortality.

Group or Phenotype	*n*	ICU Deaths, *n*	ICU Mortality, % (95% CI)
No metabolic acidosis	728	34	4.7 (3.4–6.5)
Acidosis, 0 features	74	5	6.8 (2.9–14.9)
Acidosis, 1 feature	183	12	6.6 (3.8–11.1)
Acidosis, 2 features	122	27	22.1 (15.7–30.3)
Acidosis, 3 features	29	17	58.6 (40.7–74.5)
Lactate > 2 + Cl > 108 + AG > 16	29	17	58.6 (40.7–74.5)
Lactate > 2 + AG > 16	47	19	40.4 (27.6–54.7)
AG > 16	28	6	21.4 (10.2–39.5)
Cl > 108 + AG > 16	8	1	12.5 (2.2–47.1)
Lactate > 2 + Cl > 108	67	7	10.4 (5.2–20.0)
None	74	5	6.8 (2.9–14.9)
Cl > 108	112	5	4.5 (1.9–10.0)
Lactate > 2	43	1	2.3 (0.4–12.1)
Adjusted OR per additional feature, pre-specified covariates			2.61 (2.05–3.33), *p* < 0.001
Adjusted OR per additional feature, lactate-adjusted (pooled coding)			1.82 (1.36–2.44), *p* < 0.001
Three vs. zero features, Wald	103	22	14.9 (2.4–92.2)
Three vs. zero features, Firth’s penalised	103	22	11.2 (5.8–21.6)
E-value, lactate-adjusted estimate (point/CI limit)			3.05/2.07
Adjusted OR versus no metabolic acidosis (pre-specified covariates)			
Pure lactic acidosis	43	1	0.42 (0.05–3.30)
Pure hyperchloraemic acidosis	112	5	0.86 (0.31–2.35)
Pure high anion gap acidosis	28	6	2.62 (0.87–7.89)
Overlapping (two or three features)	151	44	7.87 (4.54–13.64)
Unclassified acidosis (no feature)	74	5	1.34 (0.48–3.73)
Features entered simultaneously (within metabolic acidosis)			
Lactate > 2 mmol/L			3.17 (1.48–6.79)
Chloride > 108 mmol/L			1.89 (0.92–3.89)
Anion gap > 16 mmol/L			7.69 (3.60–16.43)
Continuous decomposition (whole cohort, primary covariates)			
Anion gap, per mmol/L			1.12 (1.05–1.18)
Strong ion difference, per mmol/L			1.01 (0.95–1.07)
Bicarbonate, per mmol/L			0.83 (0.77–0.91)

Features were lactate > 2 mmol/L, chloride > 108 mmol/L and anion gap > 16 mmol/L, counted only in patients with metabolic acidosis (base excess < −3 mmol/L) and set to zero otherwise. Phenotypes are mutually exclusive. Pre-specified covariates are age, sex, Charlson comorbidity index, admission creatinine, mechanical ventilation and non-elective surgery; admission lactate is omitted from the pre-specified specification because lactate elevation defines one of the features; the lactate-adjusted estimate is reported alongside it. Confidence intervals for crude mortality are Wilson intervals. CI, confidence interval; OR, odds ratio.

**Table 6:** ICU mortality and in-hospital mortality compared.

Measure	ICU Mortality	In-Hospital Mortality
Deaths, *n* (%)	95 (8.4)	125 (11.0)
Admission chloride spline, overall LR test	χ^2^ = 4.98, 2 df, *p* = 0.083	χ^2^ = 4.16, 2 df, *p* = 0.125
Departure from linearity	χ^2^ = 3.95, 1 df, *p* = 0.047	χ^2^ = 1.82, 1 df, *p* = 0.177
C statistic, model with chloride	0.820	0.796
C statistic, model without chloride	0.817	0.793
Adjusted OR, chloride 95 vs. 105 mmol/L	1.51 (0.72–3.19)	1.11 (0.55–2.25)
Adjusted OR, chloride 100 vs. 105 mmol/L	1.20 (0.84–1.72)	1.04 (0.74–1.46)
Adjusted OR, chloride 110 vs. 105 mmol/L	1.19 (0.96–1.48)	1.21 (0.99–1.47)
Adjusted OR, chloride 115 vs. 105 mmol/L	1.91 (1.08–3.38)	1.75 (1.04–2.93)
Adjusted OR, chloride 120 vs. 105 mmol/L	3.11 (1.18–8.19)	2.56 (1.06–6.17)
Acid-base features, adjusted OR per feature	1.82 (1.36–2.44), *p* < 0.001	1.66 (1.28–2.16), *p* < 0.001
E-value, per feature (point/CI limit)	3.05/2.07	2.71/1.88

Both columns use the identical model specification; only the outcome differs. In-hospital mortality comprises death during the index ICU admission, death during an ICU readmission within the study year, and ward death after ICU discharge. Acid–base estimates are from the covariate set that includes admission lactate as a covariate. E-values use the rare-outcome approximation. CI, confidence interval; LR, likelihood ratio; OR, odds ratio.

## Data Availability

The de-identified analysis dataset is available from the corresponding author upon reasonable request, subject to institutional data-sharing approval. The data are not publicly available because of institutional restrictions on the sharing of patient-level clinical records.

## Abbreviations

AG, anion gap; AKI, acute kidney injury; BE, base excess; CI, confidence interval; CCI, Charlson comorbidity index; ICU, intensive care unit; IQR, interquartile range; KDIGO, Kidney Disease: Improving Global Outcomes; LR, likelihood ratio; OR, odds ratio; SID, strong ion difference; STROBE, Strengthening the Reporting of Observational Studies in Epidemiology.

## Appendix A

The following tables provide supporting detail for the analyses reported in the main text.

**Table A1:** Baseline characteristics according to admission chloride category.

Variable	Hypochloraemia (*n* = 38)	Normochloraemia (*n* = 658)	Hyperchloraemia (*n* = 440)
Age, years	66 (56–78)	66 (54–74)	65 (50–73)
Charlson comorbidity index	2.00 (1.00–4.75)	1.00 (0.00–3.00)	1.00 (0.00–2.00)
Chloride, mmol/L	95 (94–97)	105 (103–107)	111 (110–113)
Sodium, mmol/L	133 (130–136)	137 (135–139)	140 (138–142)
Bicarbonate, mmol/L	24 (22–27)	23 (22–25)	22 (20–24)
pH	7.36 (7.30–7.42)	7.36 (7.32–7.41)	7.36 (7.30–7.41)
Base excess, mmol/L	0.70 (−4.42 to 2.80)	−1.30 (−3.20 to 0.70)	−2.95 (−5.33 to −0.57)
Lactate, mmol/L	1.63 (1.33–2.25)	1.62 (1.17–2.29)	1.70 (1.20–2.30)
Anion gap, mmol/L	20 (15–24)	13 (11–16)	11 (9–13)
Strong ion difference, mmol/L	43 (41–46)	36 (35–38)	33 (31–35)
Creatinine, mg/dL	1.15 (0.75–2.39)	0.88 (0.70–1.10)	0.87 (0.68–1.10)
Albumin, g/dL	3.20 (2.59–3.20)	3.40 (2.62–3.78)	2.45 (2.43–2.87)
ICU stay, days	1.48 (1.29–2.31)	1.40 (1.27–1.83)	1.41 (1.27–1.98)
Female	17 (44.7)	242 (36.8)	192 (43.6)
Non-elective surgery	11 (28.9)	170 (25.8)	134 (30.5)
Invasive ventilation	34 (89.5)	590 (89.7)	392 (89.1)
Documented acute kidney injury	5 (13.2)	17 (2.6)	14 (3.2)
ICU stay > 72 h	9 (23.7)	74 (11.2)	66 (15.0)
ICU death	9 (23.7)	48 (7.3)	38 (8.6)
In-hospital death	9 (23.7)	65 (9.9)	51 (11.6)

Data are median (interquartile range) or *n* (%). Categories are defined by admission serum chloride: <98, 98–108 and >108 mmol/L. No significance testing is presented because the groups are defined by the exposure. Albumin was available in 36 patients only (5 hypochloraemic, 17 normochloraemic, 14 hyperchloraemic); the values shown are not representative of the overall chloride categories. CCI, Charlson comorbidity index; ICU, intensive care unit.

**Table A2:** Sensitivity and robustness analyses.

Analysis	Result	Interpretation
Primary model: 3-knot spline (10/50/90th percentile: 101/107/113 mmol/L)	overall χ^2^ = 4.98, 2 df, *p* = 0.083; non-linearity χ^2^ = 3.95, 1 df, *p* = 0.047	No overall association; nominal non-linearity
Sensitivity: 4-knot spline (Harrell 5/35/65/95th: 99/106/109/115)	overall χ^2^ = 5.63, 3 df, *p* = 0.131; non-linearity χ^2^ = 4.60, 2 df, *p* = 0.100	Non-linearity is not robust to knot placement—the *p* = 0.047 finding should not be presented as established
Sensitivity: linear chloride	OR 1.13 (0.89–1.42) per 5 mmol/L, *p* = 0.312	No statistically significant linear association detected
Sensitivity: pre-specified reference (cohort median 107 mmol/L) instead of the fitted-curve minimum	risk difference 115 vs. 107 = 4.1 pp (−0.7 to 8.1)	Essentially unchanged; the contrast is not an artefact of choosing the reference from the fitted curve
Categorical chloride, adjusted	hypochloraemia OR 2.09 (0.73–5.98); hyperchloraemia OR 1.25 (0.75–2.08)	Neither category showed a statistically significant adjusted association
Adjustment for surgical field	overall chloride χ^2^ = 2.28, 2 df, *p* = 0.320	Association weakens further, consistent with case mix; the six added indicators also reduce precision
Discrimination	apparent C 0.820 (95% CI 0.780–0.879); optimism-corrected 0.807; without chloride 0.817; Brier 0.0570 vs. 0.0577	No clear incremental improvement in this cohort; the confidence interval permits a small gain
Firth’s penalised estimate, 3 vs. 0 features within the metabolic-acidosis subgroup	Wald OR 14.9 (2.4–92.2) vs. Firth OR 11.2 (5.8–21.6)	The penalised estimate remains elevated; precision is limited by the small subgroup
Per additional acid–base feature	OR 1.82 (1.36–2.44), *p* = 4.9 × e^−5^; E-value 3.05 (lower limit 2.07)	Gradient persisted across the reported sensitivity analyses; residual confounding by unmeasured severity remains possible
Decomposition: which term carries the signal	anion gap OR 1.117 (1.054–1.184) per mmol/L, *p* = 1.8 × e^−4^; SID (Na + K−Cl) OR 1.010 (0.951–1.073), *p* = 0.74; bicarbonate OR 0.833 (0.765–0.908), *p* = 3.1 × e^−5^	Hypothesis-generating pattern: the chloride-containing SID is null while the uncorrected anion gap and bicarbonate are associated. The three terms are algebraically linked, so this separates them descriptively and not mechanistically
Secondary outcome: documented AKI (*n* = 36)	χ^2^ = 0.98, 2 df, *p* = 0.611	Uninterpretable: ascertainment was from documentation only, the incidence is far below that of systematic KDIGO screening, and serial creatinine is unavailable
Secondary outcome: ICU stay >72 h (*n* = 149)	χ^2^ = 5.77, 2 df, *p* = 0.056	No association detected
Competing-risk aware composite: death or stay >72 h (*n* = 192)	χ^2^ = 12.23, 2 df, *p* = 0.002	Composite examined because death truncates length of stay; significant where neither component alone was and reported as exploratory
Restriction: elective surgery only	*n* = 821, 38 deaths; chloride overall χ^2^ = 1.43, 2 df, *p* = 0.490; per acid–base feature OR 1.93 (1.27–2.94)	Both primary conclusions unchanged
Restriction: excluding trauma and brain death	*n* = 1065, 83 deaths; chloride overall χ^2^ = 1.88, 2 df, *p* = 0.391; per acid–base feature OR 1.94 (1.41–2.66)	Both primary conclusions unchanged

Each row repeats the primary analyses under the stated variation. LR, likelihood ratio; OR, odds ratio.

**Table A3:** ICU readmissions during the study year, by indication and outcome.

Indication	Readmissions, *n*	Deaths, *n*	Mortality, %
Respiratory	26	15	57.7
Planned post-anaesthesia care	26	2	7.7
Sepsis or infection	10	7	70.0
Cardiovascular	2	2	100.0
Neurological	2	0	0.0
Renal	1	1	100.0
Not recorded	1	0	0.0
All readmissions	68	27	39.7
Ward deaths without ICU readmission	3	3	

Readmissions of patients already included in the cohort; only the first admission contributed to the primary analyses. ICU, intensive care unit.

**Table A4:** Acid–base feature count and mortality under both outcome definitions.

Group	*n*	ICU Deaths, *n* (%, 95% CI)	In-Hospital Deaths, *n* (%, 95% CI)
No acidosis	728	34 (4.7, 3.4–6.5)	50 (6.9, 5.2–8.9)
0 feature(s)	74	5 (6.8, 2.9–14.9)	7 (9.5, 4.7–18.3)
1 feature(s)	183	12 (6.6, 3.8–11.1)	20 (10.9, 7.2–16.3)
2 feature(s)	122	27 (22.1, 15.7–30.3)	31 (25.4, 18.5–33.8)
3 feature(s)	29	17 (58.6, 40.7–74.5)	17 (58.6, 40.7–74.5)

Odds ratios are adjusted for age, sex, Charlson comorbidity index, creatinine, mechanical ventilation, non-elective surgery and lactate. CI, confidence interval; ICU, intensive care unit; OR, odds ratio.

**Table A5:** Admission chloride and ICU mortality under progressive adjustment.

Model	Parameters	χ^2^ (2 df)	*p*	OR, 115 vs. 105 mmol/L
Model 0: chloride only	2	9.24	0.010	1.68 (1.04–2.70)
Model 1: + patient factors	6	7.27	0.026	2.02 (1.22–3.32)
Model 2: + urgency, ventilation, lactate	9	4.98	0.083	1.91 (1.08–3.38)
Model 3: + surgical field	15	2.28	0.320	1.53 (0.84–2.78)

Model 0, chloride spline alone; Model 1, adding age, sex, Charlson comorbidity index and creatinine; Model 2, adding non-elective surgery, mechanical ventilation and lactate; Model 3, adding surgical field. Odds ratios compare chloride 115 with 105 mmol/L. LR, likelihood ratio; OR, odds ratio.
